# The impact of primary location and age at orchiopexy on testicular atrophy for congenital undescended testis

**DOI:** 10.1038/s41598-019-45921-6

**Published:** 2019-07-01

**Authors:** Chi-Shin Tseng, Kuo-How Huang, Ming-Chieh Kuo, Chung-Hung Hong, Chung-Hsin Chen, Yu-Chuan Lu, Chao-Yuan Huang, Yeong-Shiau Pu, Hong-Chiang Chang, I-Ni Chiang

**Affiliations:** 1 Department of Urology, National Taiwan University Hospital, National Taiwan University, Taipei, Taiwan; 2 Graduate Institute of Electronics Engineering, National Taiwan University, Taipei, Taiwan

**Keywords:** Testis, Paediatric urology

## Abstract

In this study, we investigated post-orchiopexy testicular growth of undescended testes (UDTs) at different primary locations and determined the risk factors for testicular atrophy (TA). We conducted a retrospective chart review of boys who had undergone orchiopexy for UDTs during January 2001–December 2013. Patient profile, age at operation, primary UDT location, and testicular volume were noted. TA was defined as ≥50% loss of volume after orchiopexy. The primary endpoints were testicular growth and TA after orchiopexy. The secondary endpoint was risk factors for TA. In total, 182 boys had undergone regular ultrasonography; the median follow-up period was 34 months. Among 230 UDTs, 18 (7.8%) atrophic testicles were identified within a median interval of 13 months after orchiopexy. TA rates were 3.3% (1/30), 6.9% (12/173), and 18.5% (5/27) in primary suprascrotal, canalicular, and above-inguinal UDTs, respectively. The survival probability of UDT was 91%, 92% and 100% when orchiopexy was performed in age ≤1 year, 1 < age ≤2 years, and 100% in age >2 years, respectively. Multivariate analysis revealed that inguinal and above-inguinal UDTs (hazard ratio [HR] 11.76, 95% confidence interval [CI] 1.55–89.33, *p* = 0.017) and genetic or endocrine disorders (HR 3.19, 95% CI 1.19–8.56, *p* = 0.021) were the risk factors for TA, but not age at operation, premature birth, and laterality. Thus, TA incidence was higher when patients had high primary testicular locations. Early orchiopexy before two years of age may be associated with higher TA risk, while most testicles have promising growth after orchiopexy.

## Introduction

Orchiopexy is the standard intervention for congenital undescended testes (UDTs), which is recommended within the age of 6–12 months^1–4^ or in the first 18 months of life^5^. Histological analyses reveal improvements in the number of germ cells per tubule and presence of Leydig cells during early orchiopexy^2,6^. Early orchiopexy also decreases the risk of testicular cancer, preserves fertility and improves testicular growth^1,3,4^. Furthermore, the complications of orchiopexy, including recurrence, scrotal hematoma, wound infection, and vasal injury, are relatively uncommon^7^. However, testicular atrophy (TA) is a serious complication when orchiopexy is performed at the recommended age. A comprehensive review of orchiopexy literature reported that the overall risk of TA or nonscrotal positioning was approximately 15% in 1995^8^. In a recent series of 418 orchiopexies performed at a single institution, TA risk was 1.9%^9^. The rates of total TA after orchiopexy for abdominal UDT were 0–32%^10–13^.

In most studies, TA refers to the reduction in the volume observed during clinical examination compared with the recorded operative observation of size^14^. Different criteria for partial^13^ and complete^11,12^ atrophy have been reported in different studies. In addition, the measurement of the testicular volume through clinical palpation or by using an orchidometer is subjective and imprecise. In the present study, we reviewed data of patients who had undergone paediatric orchiopexy at our institution by using the appropriate criteria for TA. We delineated the primary location of UDTs and orchiopexy outcome through ultrasonography follow-up for each testicle. We also identified factors affecting the orchiopexy outcome.

## Materials and Methods

This study, including patient recruitment, waiver of informed consent, and all the study methods, was approved by the Institutional Review Board (IRB) of National Taiwan University Hospital. We retrospectively searched the electronic medical records at our institution for patients who had undergone orchiopexy because of UDTs (ICD9 752.51) between January 1, 2001, and December 31, 2013. We collected patient data, including clinical characteristics, underlying conditions, disease laterality, age at orchiopexy, serial scrotal ultrasonography results, and surgical findings. Patients with incomplete data and those aged >18 years were excluded.

### Primary UDT location

Congenital cryptorchidism is defined as the absence of testicles in the scrotal position since birth. Patients who had undergone orchiopexy because of retractile testes and exhibited acquired UDT were not included. All testes that could not be drawn down to the scrotum were documented as UDT. The lowest testicular position was finally confirmed after induction of anaesthesia. The primary locations were sorted into three major types: suprascrotal, inguinal (canalicular), and above-inguinal types. In the suprascrotal type, the testicles were located over the upper scrotum or external ring. In the inguinal type, the testicles were located in the inguinal canal. In the above-inguinal type, the testicles were located immediately above the internal ring (peeping testis), in the abdomen (on top of iliac vessels or close to kidney), or ectopic sites (superficial inguinal and suprapubic region).

### Surgical principles and techniques

When the primary location of the testicles is over the upper scrotum or external ring, orchiopexy with a scrotal approach is often selected. A single incision is made transversely at the lower scrotum. The overlying cremaster muscle is mobilised and then the testis can be drawn into the scrotum. When a major portion of the testicles is found in the inguinal canal or immediately above the internal ring, a standard inguinal approach is used. The spermatic cord is mobilised and the testis is dissected distally to the gubernacular remnant. Another transverse scrotal incision is made and the testicle is fixed within the subdartos pouch. For abdominal testes, the surgical method is decided by the surgeon based on surgeon’s expertise and length of testicular cord; it can include open transabdominal orchiopexy, laparoscopic orchiopexy, or one- or two-stage Fowler–Stephens orchiopexy.

### Measurement of testicular growth and survival and TA

We applied high-resolution ultrasonography with a linear-array transducer 7.5 and 10 MHz to measure testicle length, width, and position. A urologist confirmed the accuracy and quality of the ultrasonographic image. Seven abdominal testicles that could not be clearly found by ultrasound pre-operatively were measured soon after orchiopexy. Testicular volume was calculated using Hansen’s formula as follows^15^: testicular volume = 0.52 × length × (width)^2^. The growth ratio of a testicle was defined as the ratio of postoperative testicular volume to preoperative testicular volume. TA was defined as a ≥50% loss in postoperative testicular volume compared with the preoperative testicular volume. The testes were considered to survive when they did not meet TA criteria. The testicle survival duration extended from the date of orchiopexy to the recorded date of TA.

### Statistical analysis

All statistical analyses were performed using the SPSS statistical software (version 22.0; IBM Corp, SPSS, Inc, Chicago, IL, USA). The Mann–Whitney *U* test and Student’s *t* test were used to compare medians and means between the patient groups, respectively. Contingency tables were constructed for comparison by using the chi-square test. The Kaplan–Meier method was used for constructing survival curves. A log-rank test and Cox proportional hazard model were used to compare the testicular survival duration between the groups. A univariate and multivariate logistic regression were used for analysing risk factors; statistically significant or clinically crucial factors (*p* < 0.05 in the univariate analysis) were included in a multivariate model. All tests were two-tailed. A *p* of < 0.05 was considered significant.

### Ethical approval

The IRB of National Taiwan University Hospital approved this study (#201704024RINA). All the procedures involving human participants followed in the study were in accordance with the ethical standards of the institutional and/or national research committee and with the 1964 Helsinki declaration and its later amendments or comparable ethical standards. For retrospective studies, formal consent of the participants is not required.

## Results

We retrospectively reviewed 518 patients who had undergone paediatric orchiopexy surgery at our institution during the aforementioned study period. In total, 182 boys, comprising 134 (73.6%) and 48 (26.4%) boys with unilateral and bilateral UDT, respectively, had undergone regular ultrasonography examinations for a median follow-up period of 34 months (Table 1). The median age at operation was 14 months (interquartile range [IQR] 11.3–28.1 months). All the patients with testicles over the upper scrotum (10 testicles) and external ring (20 testicles) had undergone orchiopexy with a scrotal approach. The patients with testicles above the inguinal canal (173 testicles) and internal ring (10 testicles) had undergone orchiopexy with an inguinal approach. The patients with ectopic testes over the superficial inguinal (two testicles) and suprapubic (two testicles) regions had undergone orchiopexy with an inguinal approach. The patients with abdominal testes had undergone abdominal orchiopexy through an open inguinal approach (six testicles), laparoscopic orchiopexy (five testicles), and laparoscopic one-stage Fowler–Stephens orchiopexy (two testicles).

**Table 1:** Descriptive statistics of the primary locations and laterality of undescended testes and their procedures performed.

Number of patients	182	
Median age at orchiopexy, months (IQR)	14.1	(11.3–28.1)
Median follow-up, months (IQR)	34	(17.1–53.0)
**Primary locations of each UDT**	230	
Upper scrotum (%)	10	4.3%
External ring (%)	20	8.7%
Inguinal canal (%)	173	75.2%
Internal ring (%)	10	4.3%
Abdomen (%)	13	5.7%
Etopic testes (%)	4	1.7%
**Laterality**		
Bilateral (%)	48	26.4%
Right (%)	62	34.1%
Left (%)	72	39.5%
**Surgical procedures (each testicle)**	230	
Scrotal approach (%)	30	13.0%
Inguinal approach (%)	193	83.9%
Laparoscopic methods (%)	7	3.0%

Table 2 lists the clinical outcomes of UDTs stratified by their primary locations, namely suprascrotal, inguinal, and above-inguinal UDTs. The scrotal group, representing normally descended testes, was used as reference. The preoperative testicular volumes of all types were smaller than the volumes of normally descended testes. Furthermore, small testicular volumes were associated with high location of the UDT; the median volume of scrotal testes was 0.399 mL, whereas the median volumes of the suprascrotal, inguinal, and above-inguinal UDT were 0.302 mL, 0.229 mL, and 0.147 mL, respectively. After orchiopexy, patients with above-inguinal testes exhibited the lowest testicular volumes. However, the growth ratio in all the UDT types was higher than that in the scrotal testes (1.287). The median growth ratios of the suprascrotal, inguinal, and above-inguinal UDT were 1.418, 1.501, and 1.433, respectively.

**Table 2:** Clinical outcomes of patients with undescended testes stratified by different preoperative locations of the testicle.

	Scrotal	P Value	Suprascrotal	P Value	Inguinal	P Value	Above Inguinal	P Value
Number of patients	134		30		173		27	
Median age (IQR) at operation	13.8 (10.3–21.3)	ref.	29.2 (12.0–69.8)	0.022	14.1 (11.3–28.1)	0.444	13.8 (12.2–21.1)	0.807
Follow-up (IQR) months	34.15 (20.5–52.8)	ref.	24.6 (7.5–50.9)	0.166	34.1 (17.3–53.1)	0.87	37.2 (11.0–84.9)	0.426
**Laterality**								
Right (%)	72 (53.7%)	ref.	13 (43.3%)		84 (48.6%)		13 (48.1%)	
Left (%)	62 (46.3%	ref.	17 (56.7%)		89 (51.4%)		14 (51.9%)	
Pre-operative testicular volume (ml)	0.399 (0.292–0.533)	ref.	0.302 (0.158–0.509)	0.028	0.229 (0.133–0.350)	<0.001	0.147 (0.077–0.367)	<0.001
Post-operative testicular volume (ml)	0.516 (0.355–0.693)	ref.	0.470 (0.326–0.619)	0.260	0.322 (0.196–0.524)	<0.001	0.185 (0.109–0.527)	<0.001
Growth ratio of testicle*	1.287 (0.899–1.824)	ref.	1.418 (0.935–3.541)	0.315	1.501 (0.952–2.419)	0.044	1.433 (0.569–2.783)	0.871
Atrophy (%)	0 (0%)	ref.	1 (3.3%)	0.035	12 (6.9%)	0.002	5 (18.5%)	<0.001

*Growth ratio of testicle = Post-op testicular volume/ Pre-op testicular volume.

**Figure 1:**
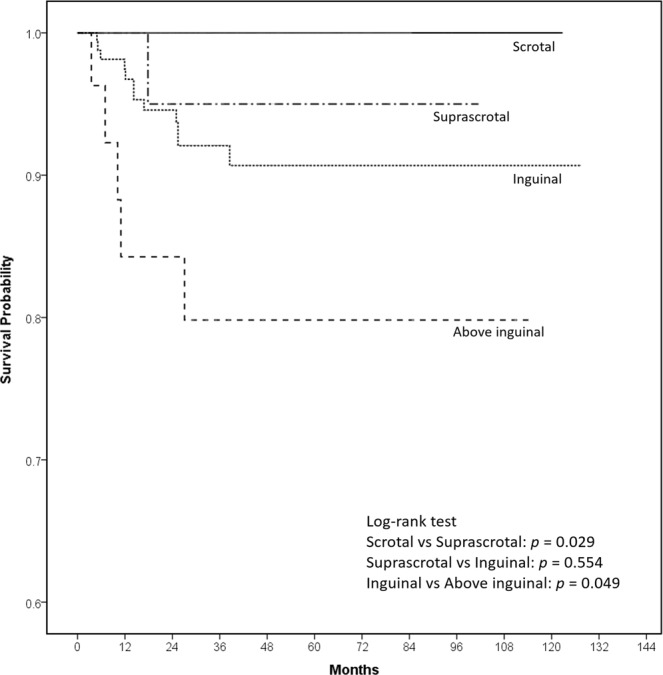
Kaplan–Meier survival curves for the survival probability of testicles in 134 normally descended testes and 230 undescended testes after orchiopexy stratified by their primary location: suprascrotal, inguinal, and above-inguinal.

Among the 230 UDTs, overall 18 (7.8%) atrophic testicles were identified after orchiopexy. TA rates were 3.3% (1/30), 6.9% (12/173), and 18.5% (5/27) in the primary suprascrotal, inguinal, and above-inguinal group, respectively (Table 2). The Kaplan–Meier survival curves revealed that the survival probability of testicles in the patients with134 normally descended testes was 100% during the follow-up period (Fig. 1). The post hoc analysis revealed that the suprascrotal UDT had a lower survival probability than the reference scrotal testes (*p* = 0.029). The survival probabilities of suprascrotal and inguinal UDTs did not differ significantly (*p* = 0.554). The survival probability of the above-inguinal UDTs was lower than that of the inguinal UDTs (*p* = 0.049). TA occurred with a median duration of 13 months (IQR 6.7–25.1 months), and only one testicle exhibited atrophy 3 years after orchiopexy.

**Figure 2:**
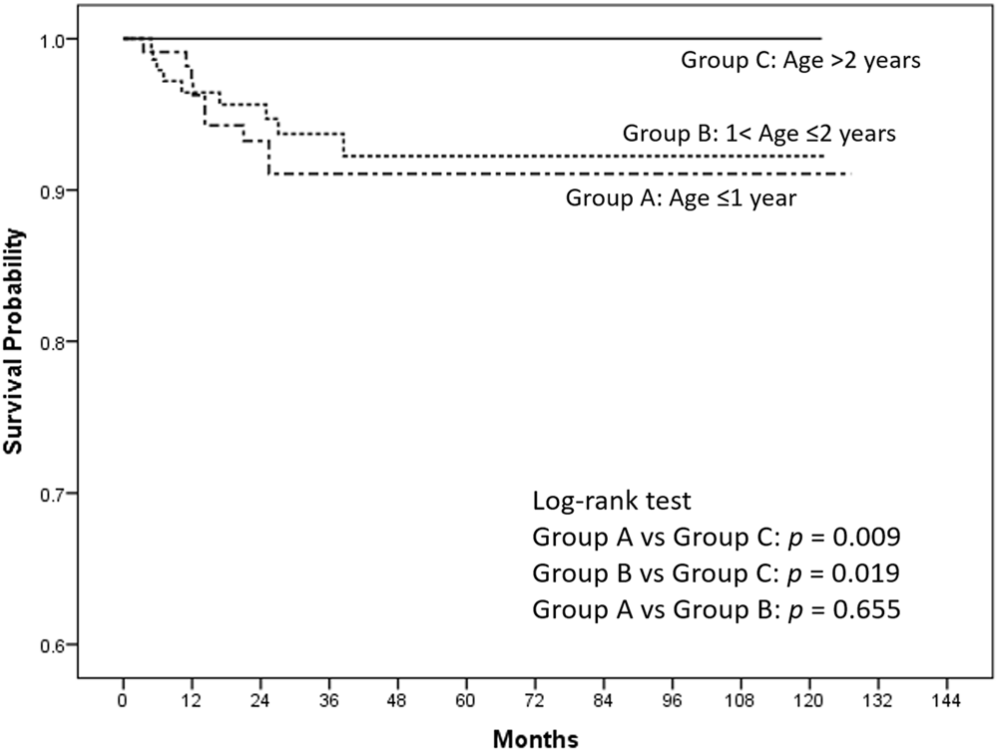
Kaplan–Meier survival curves for the survival probability of testicles in 230 undescended testicles after orchiopexy, stratified into three groups by age at orchiopexy. Groups A, B, and C: age ≤1, 1 < age ≤2, and >2 years, respectively.

Based on their age at orchiopexy, the patients were stratified into Groups A (age ≤1 year, n = 58), B (1 < age ≤2 years, n = 73), and C (age >2 years, n = 51). The Kaplan–Meier survival curves revealed the survival probabilities of 230 UDTs after orchiopexy in the different age groups (Fig. 2). The probabilities of testicular survival were 91%, 92%, and 100% in Groups A, B, and C, respectively. The post hoc analysis revealed significantly lower testicular survival probabilities in Group A than in Group C (*p* = 0.009) and in Group B than in Group C (*p* = 0.019).

The univariate logistic regression analysis (Table 3) revealed that patient age, premature birth (gestational age <37 weeks), laterality, and surgical methods were not significantly associated with TA. Moreover, the multivariate logistic regression analysis revealed that the risk factors for TA were the primary location of UDT in the inguinal and above-inguinal areas (odds ratio [OR] 11.764, *p* = 0.017) and congenital diseases (OR 3.191, *p* = 0.021). The TA risk was 15% (6 out of 40 testicles) for patients with congenital disease, including DiGeorge syndrome (2 patients), Prader-Willi syndrome (2 patients), Kallmann syndrome (1 patient), Duane’s syndrome (1 patient), Williams syndrome (1 patient), hypogonadism (4 patients), and other congenital anomalies (9 patients).

**Table 3:** Univariate and multivariate analyses of testicular survival in undescended testes.

Variables	Case number	Failure events	Univariate analysis			Multivariate analysis		
			OR	Range	P Value	OR	Range	P Value
Age	364	18	0.998	0.997–1.000	0.095	0.998	0.996–1.000	0.118
Primary location of testicles								
Scrotum + Suprascrotal	164	1	1	—	—	1	—	—
Inguinal + Above inguinal	200	17	13.91	1.851–104.52	0.011	11.764	1.549–89.326	0.017
Combined with other congenital diseases*								
No	324	12	1	—	—	1	—	—
Yes	40	6	4.496	1.687–11.983	0.003	3.191	1.190–8.558	0.021
**Premature birth or not**								
Gestation age ≥37 weeks	320	16	1	—	—	—	—	—
Gestation age <37 weeks	44	2	0.912	0.210–3.969	0.902	—	—	—
**Surgical methods**								
Scrotal approach	30	1	1	—	—	—	—	—
Inguinal approach	193	15	2.026	0.267–15.353	0.494	—	—	—
Laparoscopic approach	7	2	10.431	0.943–115.338	0.056	—	—	—
**Laterality**								
Right	182	7	1	—	—	—	—	—
Left	182	11	1.566	0.607–4.04	0.353	—	—	—

Combined with other diseases *Including DiGeorge syndrome, Prader-Willi syndrome, Kallmann syndrome, Duane’s syndrome, Williams syndrome, congenital anomalies, and hypogonadism.

## Discussion

The present study was a comprehensive analysis of the testicular growth in patients who had undergone paediatric orchiopexy. We defined TA as a volume loss of ≥50% after orchiopexy and assessed the testicular volume accurately through ultrasonography. The overall TA rate in this cohort was 7.8%. In most studies, TA refers to the reduction in testicular volume and is assessed through observation by clinical palpation^14^. However, the atrophy rate was usually underestimated because of subjective appraisal of testicular size by a single surgeon^12^. Durell *et al*. reported an overall atrophy rate of 2.6% with the same definition as that in the present study, but testicular volumes were measured through clinical palpation^16^. Ein *et al*. reported relatively high rates of TA; 5% and 9% in the common and high UDT type, respectively, and the definition used was a decrease in size of the testicle by at least one-third^17^. Several studies have reported various criteria with an atrophy rate of up to 32%^11,13,18^.

The median duration from orchiopexy to TA was 13 months (IQR 6.7–25.1 months). Alternatively, most TA occurred within the first 2 years after orchiopexy. In particular, in patients with above-inguinal UDTs, abdominal testes and peeping testes exhibited atrophy within a median duration of 10.2 months, which was considered relatively early. The TA rate was high when the primary UDT locations were high. In the present study, the atrophy rates were 3.3%, 6.9%, and 18.5% in patients with suprascrotal, canalicular, and above-inguinal canal UDTs, respectively. The UDT outcomes were superior to those reported in a literature review in 1995 about varying success rates for orchiopexy, namely 74% for abdominal UDTs, 82%–87% for inguinal UDTs, and 92% for UDTs at the level of the external ring^8^.

TA refers to the decrease in the size of the testes and concomitant loss in testicular function. The aetiologies of primary TA include congenital genetic disorders, such as Klinefelter syndrome and Kallmann syndrome, sexual development disorders, hormonal regulation disorders, and history of ischaemic episodes, such as intrauterine or prepubertal testicular torsion. Nevertheless, the testicular volume in patients with UDTs before orchiopexy is significantly lower at the age of 6 months compared with the normal control group^19,20^. Patients with congenital diseases (OR 3.191, *p* = 0.021) had a higher TA risk than those without congenital diseases in the multivariate analysis in this study. Therefore, some congenital factors other than the vascular ischaemic episodes affect the testicular growth in UDTs. Whether primary TA is a risk factor for testicular tumour development, which requires orchiectomy, remains unknown.

Secondary TA is caused by varicocele in adolescence, scrotal trauma, infection, and surgical repairs of inguinal hernias. TA in UDTs after orchiopexy tends to be secondary in most cases. In present study, the TA occurred within a median interval of 13 months after orchiopexy. If the UDT survived after orchiopexy, the testicular growth ratio was superior to the normally descended testes in all primary location groups and age groups. TA after orchiopexy may result from the following causes: tension in the spermatic cord with subsequent testicular ischaemia, torsion of or injury to the spermatic cord when passing the testes to the scrotum, insufficient collateral vessels after ligation of the spermatic cord in Fowler–Stephens orchiopexy, and testicular tissue loss due to the strangulation by the surgical string.

Before 2006, only two studies in the review revealed a median age at orchiopexy <2 years^18^. A large population study in New South Wales, Australia, revealed that the median age at surgery was 16.6 months (IQR 11.8–31.0 months) and two-thirds of orchiopexies were performed after the age of 12 months from 2001 to 2011^21^. In the present study, the median age at operation was 14 months (IQR 11.3–28.1 months). The patients who underwent orchiopexy for inguinal and above-inguinal UDTs were younger than those for suprascrotal UDTs. For nonpalpable testes, surgeons recommend orchiopexy at an early age for preserving fertility later in life. In addition, the distance over which a surgeon will need to draw the testes into the scrotum increases with the longitudinal growth of a patient. Physician tends to wait and see at first for suprascrotal UDTs that procrastinates the timing of orchiopexy. Sometimes, the tethered testes initially descend and become higher in position when the patient grows.

Orchiopexy should be performed between 6 and 12 months of age according to a Nordic consensus^22^. UDTs are associated with progressive germ cell loss. Tasian *et al*. reported a higher germ cell depletion risk in nonpalpable testes, including intra-abdominal testes (45 of 102 testes), than in palpable testes^6^. Many studies have revealed high testicular growth after treatment of UDT with early orchiopexy^4,23^. However, paediatric orchiopexy in such a young age requires appropriate technique and experience, or any treatment failure can cause distress. Carson *et al*. reported an opposite outcome that the TA rate was lower when orchiopexy was performed before the age of 13 months (TA rate: 5%) than when it was performed at the ages of 13–24 months (TA rate: 12%)^24^. In our study, the atrophy rate for orchiopexy before 1 year of age (9%) was noninferior (*p* = 0.655) to that for orchiopexy at ages of 1–2 years (8%). Nevertheless, no TA was observed in patients who underwent orchiopexy after 2 years of age. This phenomenon could be explained by the higher strength of the spermatic cord and better developed collateral blood supply in the testis in the patients who were >2 years of age than in those who were <2 years of age. However, orchiopexy after 2 years of age is not recommended because clinical and pathological evidence has shown that the overall outcomes of the testes were inferior to those of orchiopexy at <2 years of age.

In a previous study, the preoperative location of UDT did not affect the final changes of testicular volume in patients that received pre-operative hormone therapy^25^. However, the primary location of UDTs actually had an impact on the testicular volume and TA rates in this study. Besides, hormone therapy is an abandoned management nowadays. Although the primary locations have an impact on testicular volume after orchiopexy, the growth ratio in all the UDT types was high.

The present results also simplified the use of the TA index (TAI), which is used in the assessment of surgical outcomes of varicocele^26^. TAI is an objective tool of qualifying patients with UDT for surgery as well as monitoring surgical outcomes^27^. The TAI (expressed as a percentage) is calculated as follows: TAI = (contralateral testis volume − affected testis volume)/contralateral testis volume × 100. For patients with bilateral testicular diseases, a normalised TAI is calculated as follows^28^: (normative value of testis − affected testis volume)/normative value of testis. The normative value of testicular volume for each age group were measured through ultrasonography in a normal population by Goede *et al*.^29^. The testicular growth percentage is calculated as follows: postoperative testis volume/preoperative testis volume × 100. The TA was identified as a testicular growth percentage <50%.

Our study had several advantages, such as a large number of patients who had undergone paediatric orchiopexy, accurate measurement of testicular volumes through ultrasonography, and regular follow-up after surgery. However, we should carefully interpret the results while applying them to clinical practice. The limitations include the retrospective nature of this study that caused an intrinsic bias and the heterogeneity of patient groups. Although we collected all our data carefully, we could not retrieve several missing data; consequently, we excluded patients without complete data. Furthermore, urologists and paediatric surgeons might have different levels of experience and may follow different techniques, particularly while treating abdominal testes. Furthermore, prospective data registry and standard protocols are required to investigate TA risks and address the safety concerns associated with early orchiopexy.

In conclusion, UDTs exhibited relatively low volumes and higher atrophy rate when the primary location of the testicle is high. Nevertheless, early orchiopexy is recommended because the testicular growth of UDTs is promising and faster than normally descended testis if the UDTs survive after orchiopexy. In general, higher TA risk was observed when orchiopexy was done within 2 years of age and when patients had high primary UDT locations. We should be aware of TA risk while performing early orchiopexy at such a young age.
